# Circulating Ionized Magnesium as a Measure of Supplement Bioavailability: Results from a Pilot Study for Randomized Clinical Trial

**DOI:** 10.3390/nu12051245

**Published:** 2020-04-28

**Authors:** Jiada Zhan, Taylor C. Wallace, Sarah J. Butts, Sisi Cao, Velarie Ansu, Lisa A. Spence, Connie M. Weaver, Nana Gletsu-Miller

**Affiliations:** 1 Public Health Nutrition, Case Western Reserve University, 10900 Euclid Avenue, Cleveland, OH 44106, USA; jxz1119@case.edu; 2 Department of Nutrition and Food Studies, George Mason University, MS1F7, 4400 University Drive, Fairfax, VA 22030, USA; 3 Think Healthy Group, Inc., 1301 20th Street NW, Washington, DC 20036, USA; 4 Center for Magnesium Education & Research, 13-1255 Malama Street, Pahoa, HI 96778, USA; 5 Department of Nutrition Science, Purdue University, 700 West State Street, West Lafayette, IN 47907, USA; sjb1602@gmail.com (S.J.B.); caosisi0703@gmail.com (S.C.); 6 Department of Applied Health Science, School of Public Health, Indiana University Bloomington, Bloomington, IN 47405, USA; vansu@iu.edu (V.A.); lisspenc@iu.edu (L.A.S.); ngletsum@indiana.edu (N.G.-M.); 7 Weaver and Associates Consulting, LLC, West Lafayette, IN 47906, USA; weaverconnie1995@gmail.com

**Keywords:** magnesium, iMg, biomarkers, nutritional status, diet

## Abstract

Oral supplementation may improve the dietary intake of magnesium, which has been identified as a shortfall nutrient. We conducted a pilot study to evaluate appropriate methods for assessing responses to the ingestion of oral magnesium supplements, including ionized magnesium in whole blood (iMg^2+^) concentration, serum total magnesium concentration, and total urinary magnesium content. In a single-blinded crossover study, 17 healthy adults were randomly assigned to consume 300 mg of magnesium from MgCl_2_ (ReMag^®^, a picosized magnesium formulation) or placebo, while having a low-magnesium breakfast. Blood and urine samples were obtained for the measurement of iMg^2+^, serum total magnesium, and total urine magnesium, during 24 h following the magnesium supplement or placebo dosing. Bioavailability was assessed using area-under-the-curve (AUC) as well as maximum (C_max_) and time-to-maximum (T_max_) concentration. Depending on normality, data were expressed as the mean ± standard deviation or median (range), and differences between responses to MgCl_2_ or placebo were measured using the paired *t*-test or Wilcoxon signed-rank test. Following MgCl_2_ administration versus placebo administration, we observed significantly greater increases in iMg^2+^ concentrations (AUC = 1.51 ± 0.96 vs. 0.84 ± 0.82 mg/dL•24h; C_max_ = 1.38 ± 0.13 vs. 1.32 ± 0.07 mg/dL, respectively; both *p* < 0.05) but not in serum total magnesium (AUC = 27.00 [0, 172.93] vs. 14.55 [0, 91.18] mg/dL•24h; C_max_ = 2.38 [1.97, 4.01] vs. 2.24 [1.98, 4.31] mg/dL) or in urinary magnesium (AUC = 201.74 ± 161.63 vs. 139.30 ± 92.84 mg•24h; C_max_ = 26.12 [12.91, 88.63] vs. 24.38 [13.51, 81.51] mg/dL; *p* > 0.05). Whole blood iMg^2+^ may be a more sensitive measure of acute oral intake of magnesium compared to serum and urinary magnesium and may be preferred for assessing supplement bioavailability.

## 1. Introduction

Magnesium is an element with an atomic number of 12 and a mass of 24.32 Da. It is the fourth most abundant mineral in the human body, with > 99% residing in the bone, muscle, and nonmuscular soft tissue and < 1% residing in the serum and red blood cells [1,2]. Magnesium is also the second most abundant intracellular cation [3]. Enzymatic databases list over 600 enzymes for which magnesium serves as a cofactor, and an additional 200 in which it may act as an activator [4,5,6]. The intracellular level of free magnesium ions regulates intermediary metabolism, DNA and RNA synthesis and structure, cell growth, reproduction, and membrane structure. Thus, the cation exerts numerous physiological functions, including control of neuronal activity, cardiac excitability, neuromuscular transmission, muscular contraction, vasomotor tone, blood pressure, and peripheral blood flow [3]. Magnesium also plays a role in the movement of sodium and potassium across membranes [7]. The antiarrhythmic potency of magnesium has been described repeatedly since 1935, both as a factor in human disease and animal experiments [8,9].

Substantial portions of the U.S. population fail to meet dietary recommendations for magnesium [10,11]. Approximately 48% of the U.S. population consumes less than the estimated average requirement [12]. Furthermore, racial or ethnic differences in magnesium intake exist and may contribute to some health disparities [10]. The top 10 contributors to dietary magnesium intake in the United States are plant-based protein foods (9.52%); breads, rolls, and tortillas (7.42%); coffee and tea (7.03%); vegetables, excluding potatoes (6.12%); plain water (4.56%); milk (4.23%); fruits (4.20%); ready-to-eat cereals (3.68%); mixed dishes (meat, poultry, fish) (3.56%); and grain-based mixed dishes (3.25%) [13]. Decreased intake can result from inadequate dietary consumption, starvation, and alcohol dependence. Hypomagnesemia is an electrolyte disturbance that is caused by magnesium deficiency and is clinically defined as a serum total magnesium concentration more than 2 standard deviations below the mean of the general population [14,15]. Causes include low dietary intake, alcoholism, diarrhea, increased urinary loss, poor absorption from the gut, and diabetes mellitus [4,16]. Some medications, including proton pump inhibitors and furosemide [17], also cause low magnesium. The prevalence of hypomagnesemia is higher (11%–48%) in those with diabetes mellitus [15,18]. Hypomagnesemia is common in hospitalized patients (20%) [19] and is even more frequent in patients with other coexisting electrolyte abnormalities [20,21,22] and in critically ill patients (20%–65%) [23,24].

Many studies have focused on the measurement of serum total magnesium concentration because of ease of measurement rather than its free bioactive iMg^2+^ form, making it difficult to correlate to disease states [25] or to truly assess status. Estimation of the iMg^2+^ levels in serum or plasma by analysis of ultrafiltrates (complexed magnesium + iMg^2+^) is better than total magnesium measures, but it does not distinguish the truly ionized form from that which is bound to organic and inorganic anions [25]. Technological advances have improved the reliability for measuring iMg^2+^ in complex matrices such as whole blood, plasma, and serum by improving the durability of the ion-selective electrodes and by reducing the interference on the electrode from other cations such Ca^2+^ [26]. Given the capacity for rapid, accurate, and reliable analysis of electrolytes, commercially available analyzers are now routinely used in the clinical setting [26,27]. However, these instruments have rarely been used for research purposes. An early bioavailability study used ion-selective electrode technology to measure iMg^2+^ responses in serum following administration of magnesium oxide formulations [28]. However, the study participants ingested high magnesium diets beforehand, creating magnesium-saturated individuals who are not a representative sample of the population. Our research group is currently evaluating the bioavailability of ReMag^®^, a formulation of MgCl_2_ against a commonly marketed Mg supplement, MgO, which is often used for bioavailability studies [28,29,30]. We are utilizing a randomized clinical study design and newer technology with analytical advances in the assessment of iMg^2+^ to test acute oral doses of these two supplements against a placebo. In the pilot study reported here, our objective was to determine the utility of whole blood concentrations of iMg^2+^, compared to concentrations of serum total magnesium and total urinary magnesium, as a biomarker of response to an oral challenge consisting of a single dose of magnesium chloride, among healthy individuals. By evaluating the acute bioavailability of a one-time dose of ReMag^®^, a novel formulation of MgCl_2_, our results may be extrapolated to assess the feasibility of this formulation to increase iMg^2+^ status. This pilot study provided critical learnings for the current study and both studies will be useful for any future study assessing chronic administration of magnesium supplements. Future work will investigate effects of longer-term dosage of magnesium supplementation on magnesium status and markers of disease risk.

## 2. Materials and Methods

The main study will be a randomized, single-blind, placebo-controlled crossover trial that aims to compare acute pharmacokinetics following ingestion of 300 mg (12.34 mmol) of magnesium from ReMag^®^ (a formulation of MgCl_2_) against MgO. We describe the similarly designed pilot study here, which aimed to evaluate the methodology for assessing magnesium and to generate data to inform sample size calculations for the main study. For the pilot study, we compared a formulation of magnesium chloride (MgCl_2_) called ReMag^®^ (New Capstone, Inc., Mooresville, NC, USA) to a vehicle placebo of water (and lemon juice to mask the taste of the MgCl_2_ formulation). Using a molecular size analyzer (Malvern Zetasizer Nano ZS, Malvern, UK), we confirmed that the majority of the particle size of ReMag^®^ formulation was in the picometer range (52% was 800 pm and 48% was 2.5 μm); we speculated that the small particle size would promote rapid absorption. Using atomic absorption spectrometry (5100 PC; Perkin-Elmer, Waltham, MA, USA), we determined that the concentration of magnesium in the ReMag^®^ formulation was 44.73 mg/g of sample and this concentration was used to deliver a dose of 300 mg of magnesium from the ReMag^®^ formulation. The study protocol was approved by the Purdue University Institutional Review Board (protocol number 1802020279) and is registered at ClinicalTrials.gov (NCT04139928). 

### 2.1. Study Population 

Recruitment was conducted via flyers, emails, and word-of-mouth. Prior to the screening visit at the clinic, interested volunteers were contacted by study staff and prescreened using a medical questionnaire. The medical questionnaire included questions related to participants’ medical history (occurrence of any cardiac, digestive, renal, and other diseases, i.e., thyroid disorder, diabetes, and cancer). Other questions included recent blood donation, intake of Mg supplements, pregnancy or intention to become pregnant (females only), and medications that participants were currently using. Potential participants were then mailed a consent form and asked to complete a 3-d food log to capture habitual dietary intake before their screening visit. Participants fasted overnight prior to the screening visit. Patients provided written informed consent before entering the study.

Healthy adult men and women of all races/ethnicities, aged 18–65 years, with a body mass index (BMI) of 18–35 kg/m^2^ could enter the study after the screening visit. Individuals were excluded if they met any of the following criteria: (1) had a diagnosis of hypertension, prehypertension, diabetes, cardiovascular disease, or other chronic diseases (e.g., cancer); (2) had been diagnosed with hypermagnesemia (> 2.28 mg/dL); (3) had been diagnosed with gastrointestinal disease, hepatitis, anemia, or hepatic enzyme abnormalities or were currently taking magnesium supplements or medications that interfere with magnesium absorption or metabolism within 2 wk of screening; (4) were currently pregnant or trying to become pregnant; (5) had a history of hospitalization for acute illness within 1 month prior to screening; (6) were unable to speak English or were unable able to comprehend the informed consent; (7) had a habitual diet which contained an excess of high magnesium foods (by reviewing dietary intake from 3-day food logs prior to the screening visit); or (8) were unable or failed to complete the full medical questionnaire. Participants were financially compensated for their participation in the study.

### 2.2. Magnesium Dosing

Enrolled participants (Males = 7, Females = 10, Total *N* = 17) were randomly assigned to a single-dose treatment of MgCl_2_ (ReMag^®^; in a solution of lemon juice and water) or placebo (lemon juice and water only) with a low-magnesium (~50 mg Mg) breakfast after 8 h of fasting. Participants were blinded to their treatment assignment, but the research team was not blinded. Participants partook in two clinic visits with a minimum of a 7-d washout period between treatments. A low-magnesium lunch, dinner, and evening snack, designed by a research dietitian to contain < 160 mg of magnesium, and low-mineral water (Aquafina; PepsiCo Inc., Purchase, Harrison, NY, USA) were also provided on the days of the clinical visits. Participants were asked to refrain from consuming foods and beverages, aside from those provided, on the days they visited the clinic. Examples of foods and beverages served during participants clinic visit days were: (1) breakfast—omelet with eggs and vegetables (peppers and zucchini) and water or apple juice; (2) lunch—stir fry with rice and vegetables (cabbage, zucchini, carrots); (3) dinner—rice noodle stir fry with vegetables (carrots, celery, peppers) and (4) evening snack—vanilla ice cream. Lunch and dinner were provided to coincide with the 4- and 8-h sample collection periods. After the 8-h sample collection, participants were given their evening snack and water, allowed to leave the clinic, and asked to return the next morning for the 24-h sample collections. 

### 2.3. Specimen Collection and Measurements 

Blood samples were obtained beginning with a fasting sample (at 15 min before the dosing) and at 0, 0.5, 1, 2, 4, 6, 8, and 24 h following the dosing. The timepoints chosen are standard for bioavailability tests to assess serum concentrations [28,31]. Specimens were collected within ± 15 min of the hourly time points and within ± 30 min of the 24-h timepoint. Venous blood samples were collected in lithium heparinized tubes and in serum separator tubes for measurement of iMg^2+^ and serum total magnesium concentrations, respectively. 

The whole blood concentration of iMg^2+^ was determined using previously described standardized methods [32] with a Nova 8 Electrolyte Analyzer (Nova Biomedical, Waltham, MA, USA). The instrument is designed as a point-of-care analyzer for the critical care setting, and it is rapid and easy to use along with automated quality control. Previous studies reported that iMg^2+^ was fairly stable for at least 6 h when stored in capped lithium heparinized tubes at either room temperature or 4 °C [26,33]. However, our in-house testing suggested that the whole blood iMg^2+^ level was relatively stable when stored at 4 °C for over 4 h, but there was a mean decrease of 7.14% after storing at room temperature for 2 h. Thus, to ensure accuracy and consistency, blood samples were measured within 10 min of collection. The intra- and inter-day coefficient of variation (CV) values for iMg^2+^ were less than 3%, as reported previously [26]. 

Serum was separated from whole blood samples and frozen before analysis. Urine specimens were collected from participants 15 min before dosing and the total urine collected at 2, 4, 6, 8, 8–21, and 24 h post dosing were pooled in batches. For each urine sample, the specific gravity was measured to determine the urine concentration and the hydration status, and the sample was frozen before analysis. Concentration of serum total magnesium and total urinary magnesium content were determined by atomic absorption mass spectrometry using previously described standardized methods [34]. All CVs for each serum and urine magnesium measurement were below 4%. 

### 2.4. Statistical Analyses

We did not conduct power analysis for the pilot study because we intended for the pilot data to inform the sample size of the main study, but the size of our sample compares to a similar bioavailability study by Altura et al. [28]. We analyzed the 24-h area under the curve (AUC) to obtain the maximum concentration (C_max_) and the time to maximum concentrations (T_max_) for each of the iMg^2+^ and serum total magnesium concentrations and the total urinary magnesium content responses using OriginPro software (2019 version; OriginLab, Northampton, MA, USA). Data were analyzed in terms of descriptive statistics and tested for normality (Z-skewness cutoff = 1.96). The mean ± standard deviation (SD) was used to report data with a normal distribution. When the data were not normally distributed, we reported the median and range. Differences between MgCl_2_ and placebo responses were compared using paired *t*-tests, or the nonparametric equivalent, Wilcoxon signed-rank test, based on the normality of the groups. To access the impact of confounding variables such as age, baseline serum total magnesium, or BMI on iMg^2+^, serum total magnesium, or urine magnesium measures, we conducted partial correlations as well as linear regression. A *p* value of 0.05 was used to determine statistical significance. IBM SPSS software (version 26; IBM, Armonk, NY, USA) was used for all statistical analyses.

## 3. Results

**Figure 1:**
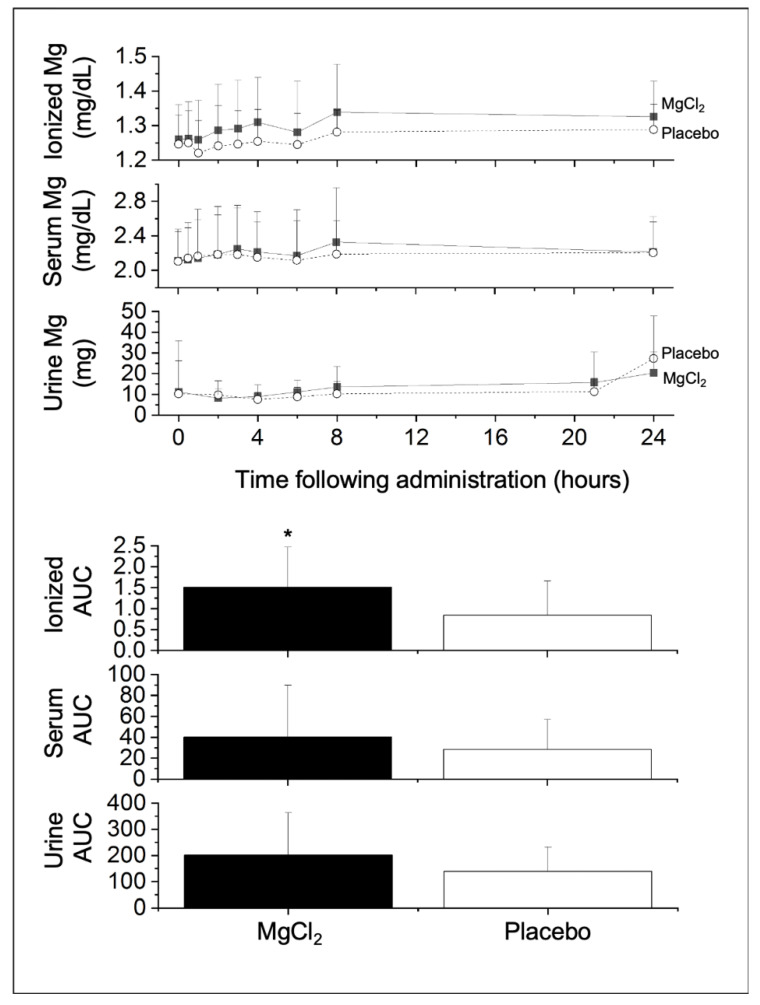
Average whole blood concentration of ionized magnesium (iMg^2+^) and serum total magnesium concentrations, as well as urinary magnesium content (top); total area under the curve (AUC) in mg/dL•24h for iMg^2+^ and serum total Mg or mg•24h for urine Mg (bottom) over 24 h post-treatment for MgCl_2_ treatment vs. placebo. * Differences between MgCl_2_ versus placebo, *p* < 0.05. To convert between mg/dL and mmol/L, divide by 2.43.

A total of 17 participants were enrolled in the pilot study and their baseline characteristics are presented in Table 1. These participants were young adults (median age 25 y [18–44 y]), with normal weight (mean BMI, 24.7 ± 3.7 kg/m^2^), slightly more female (58.8%) than male (41.2%) participants, and representation from various racial and ethnic groups. We excluded from the data analysis participants who had data missing for the iMg^2+^, serum, or urinary magnesium assays after administration of MgC1_2_ or placebo; thus, we collected complete matched pairs data with 14 participants for iMg^2+^, 17 participants for serum total magnesium, and 12 participants for urinary magnesium. Graphs of the absorption curves and total AUC of iMg^2+^, serum, and urinary magnesium measures over 24-h post-treatment of MgCL_2_ vs. placebo can be found in Figure 1. 

Following administration of MgCl_2_, we found a significant greater increase in the 24-h AUC of whole blood concentration of iMg^2+^ (1.51 ± 0.96 mg/dL•24h) compared to that increase after administration of placebo (0.84 ± 0.82 mg/dL•24h) (*p* = 0.029, Table 2). We also observed a greater increase in C_max_ for iMg^2+^ between MgCl_2_ (1.38 ± 0.13 mg/dL) vs. placebo (1.32 ± 0.07 mg/dL) (*p* = 0.034). There was no difference in T_max_ for iMg^2+^ between MgCl_2_ vs. placebo. We found no difference in AUC between MgCl_2_ vs. placebo for serum total magnesium (*p* = 0.097) or for urinary magnesium (*p* = 0.118). Similarly, no differences in C_max_ and T_max_ were found between MgCl_2_ vs. placebo for serum or urinary magnesium. In linear regression analyses, with AUC of iMg^2+^, serum total magnesium, and urine magnesium as the dependent measures, and age, serum total magnesium at screening, or BMI as independent measures in the model, we did not find any significant coefficients, suggesting that none of these factors were confounders.

## 4. Discussion

In this pilot study, we demonstrated the superiority of concentrations of iMg^2+^ in blood, compared to concentrations of total magnesium in serum and total urine magnesium content, as a rapid and sensitive measure of dietary intake of magnesium in healthy humans. The finding that a single dose of 300 mg of magnesium can alter iMg^2+^, but not total serum total magnesium, suggests that the iMg^2+^ method is more sensitive and reflects a subject’s dietary intake. 

Our findings of a more robust iMg^2+^ response, when compared to other magnesium biomarkers, are similar to those reported by Altura et al. [28], when measurement of iMg^2+^ using ion-selective electrodes was new. In their study, they similarly administered a 300-mg dose of magnesium, but they did not have a placebo comparator; instead, participants were randomized to three different formulations of magnesium oxide. The MgO used in the previous study is known to have low bioavailability compared to the MgCl_2_ formulation used in the current study [37]. In the current study, we also show the importance of having a placebo, which enabled us to observe circadian fluctuations of iMg^2+^ as well as of serum or urinary magnesium. Another difference between the previous study by Altura et al. and the current study was that we measured iMg^2+^ in whole blood that was freshly obtained, whereas the previous study measured iMg^2+^ in frozen plasma. Accurate and precise measurement of iMg^2+^ in blood has only recently been available in clinical practice and for research purposes. The use of more precise ion-selective electrodes allows one to make measurements of whole blood iMg^2+^ within minutes. Given that iMg^2+^ readings change over time of storage, measures taken from freshly obtained whole blood are likely more accurate and reliable [33]. Differences notwithstanding, our current study and the previous study by Altura et al. support the utility of iMg^2+^ in bioavailability studies for measuring the impact of acute oral administration of magnesium. To determine whether a more rapid response, due to a potentially enhanced bioavailability of the MgCl_2_ formulation (ReMag^®^), will be observed compared with another magnesium formulation (MgO) is an objective for the main study.

Other studies have examined the pharmacokinetics of the various Mg formulations. Blancquaert et al. conducted an in vivo study that aimed at evaluating methods that could be used in assessing Mg absorption after a single acute dosing of supplement (Ultractive Mg with 392 mg of elementary Mg vs two placebos) over six hours using blood and urine for the analysis. Results showed a significant difference for the absorption of serum total magnesium after four hours of supplement versus placebo ingestion as well as significant differences in total AUC for serum Mg. In a study by Rooney et al. [29], of chronic magnesium supplementation for 10 weeks at 400 mg magnesium per day via MgO, the findings were greater increases in both iMg^2+^ concentrations in whole blood, and serum total magnesium concentrations after Mg supplementation as compared to placebo.

In the current study, because we only assessed iMg^2+^ after an acute small oral dose of magnesium, we did not evaluate whether iMg^2+^ is an appropriate measure of magnesium status. In the crossover, a randomized clinical trial by Rooney in which healthy participants received 10 weeks of chronic supplementation of MgO versus placebo, both fresh blood concentration of iMg^2+^ and total circulating magnesium concentrations increased [29]. Although serum magnesium is used more often to assess status, the relationship between serum total magnesium and iMg^2+^ in health and chronic disease has not been extensively studied. As subclinical magnesium deficiency can occur when serum total magnesium levels are within the lower reference range [38], considerations based solely on serum total magnesium levels may not be sufficient for diagnosis of deficiency. Thus, typical symptoms and data from patient history are critical when evaluating magnesium status and the diagnosis of magnesium deficiency has therefore been based on three criteria: 1) clinical symptoms, 2) patient history, and 3) laboratory analysis of serum samples [39]. Since free ionized magnesium is considered as metabolically active, assessment of whole blood iMg^2+^ makes sense from a biological perspective. It may be important to screen for magnesium status in various populations who may be at risk due to low dietary intake, underlying diseases, or genetic susceptibility. Clinical investigations have demonstrated that iMg^2+^, but not serum total magnesium, is depressed in a number of clinical conditions such as in patients with migraine, individuals with noninsulin-dependent diabetes, patients with asthma, and women with high-risk pregnancies [28]. Magnesium homeostasis is likely to be affected by common genetic variants, similarly to most nutrients. A recent investigation found associations between genetic variations in magnesium-related ion channel genes and type 2 diabetes risk in both African American and Hispanic American women [40]. Prior to the revision of the 1998 Dietary Reference Intakes, we strongly advocate for increased government funding to assess whether whole blood iMg^2+^ better reflects the magnesium requirement in humans.

Our pilot study has some limitations. We did not exclude participants who were smokers, which may impact the assessment of iMg^2+^, as thiocyanate, a product of smoking, interferes with the Nova 8 magnesium sensor [41]. We expect this issue to be minimal since our university campus, like many others, participates in a smoke-free policy [42]. We monitored participants’ typical dietary intake before the start of the study but did not control the participants’ dietary intake of magnesium during the study, except during testing of the acute dose of magnesium. However, even if participants consumed high intakes of magnesium prior to either the placebo or MgCl_2_ on study days, we do not expect the background diet to impact response to the Mg supplement, given the lack of relationship between baseline serum total Mg and the iMg^2+^, serum Mg, and urine Mg responses. Additionally, the study conducted by Altura et al. [28] was done in individuals who were Mg loaded, yet they were able to observe an increase in iMg^2+^ after administration of the Mg dose. A larger sample size may have resulted in differences in serum total magnesium concentration and total urinary magnesium after an acute dose of magnesium compared to placebo. There is a possibility of acidosis following MgCl_2_ administration which would promote urinary Mg loss. We did not measure blood pH in this study. However, to address potential issue of acidosis we will obtain information on blood and urine pH from the ionized selective electrode during the main study. Thus, each of the limitations of the pilot study will be overcome in the main study.

## 5. Conclusions

Whole blood concentration of iMg^2+^ may be a more sensitive indicator of an acute response to a magnesium load compared to concentrations of serum total magnesium and total urinary magnesium. To use iMg^2+^ as a valid nutritional biomarker or a biomarker of magnesium status, it is essential to establish a reliable reference range in a healthy population with a large sample size. This work is currently underway.

## Figures and Tables

**Table 1:** Baseline characteristics of the study participants (*N* = 17).

Characteristic	Value ^1^	Reference Range
Age (y)	25.0 (18–44)	
Male (%)	41.2	
Female (%)	58.8	
Race/ethnicity (%)		
White	58.8	
Black	11.8	
Hispanic	5.9	
Asian	23.5	
Body mass index (kg/m^2^)	24.7 ± 3.7	
iMg^2+^ (mmol/L)	0.52 ± 0.03	0.44–0.59
Serum total magnesium (mmol/L)	0.84 ± 0.01	0.75–0.95
Serum creatinine (mmol/L)		
Female (mmol/L)	0.30 ± 0.07	0.23–0.42
Male (mmol/L)	0.37 ± 0.05	0.30–0.49

^1^ Values are given as the mean ± SD or the median (range) depending on the normality of the data. The following citations provided reference ranges: iMg^2+^ [33]; serum total magnesium [35]; serum creatinine [36].

**Table 2:** Absorption kinetics for whole blood iMg^2+^ and serum total magnesium concentrations and urinary magnesium content.

Parameter	Placebo	MgCl_2_	*p* Value
iMg^2+^ (*n* = 14)			
Baseline (mg/dL)	1.25 ± 0.08	1.26 ± 0.01	0.242
AUC (mg/dL•24h)	0.84 ± 0.82	1.51 ± 0.96	0.029
C_max_ (mg/dL)	1.32 ± 0.07	1.38 ± 0.13	0.034
T_max_ (h)	9.54 ± 9.85	10.36 ± 9.30	0.396
Serum magnesium (*n* = 17)			
Baseline (mg/dL)	1.95 (1.78–3.30)	2.08 (1.76–3.19)	0.491
AUC (mg/dL•24h)	14.55 (0–91.18)	27.00 (0–172.93)	0.097
C_max_ (mg/dL)	2.24 (1.98–4.31)	2.38 (1.97–4.01)	0.221
T_max_ (h)	11.38 ± 9.93	10.82 ± 9.11	0.396
Urine magnesium (*n* = 12)			
Baseline (mg)	2.90 (0.81–47.15)	2.95 (0.35–88.63)	0.267
AUC (mg•24h)	139.30 ± 92.84	201.74 ± 161.63	0.118
C_max_ (mg)	24.38 (13.51–81.51)	26.12 (12.91–88.63)	0.469
T_max_ (h)	24.00 (0–24.00)	24.00 (0–24.00)	0.363

Whole blood concentration of iMg^2+^, concentration of total magnesium in serum, and total content of magnesium in urine collected during 24 h of MgCl_2_ versus placebo administration are shown. Values are given as the mean ± SD or the median (range) depending on the normality of the data. AUC = the area under the curve, C_max_ = maximum (or peak) concentration, and T_max_ = time (in hours) at which C_max_ is observed. One-sided *p* values are shown.

## References

[1] Newhouse I.J., Finstad E.W. (2000). The effects of magnesium supplementation on exercise performance. Clin. J. Sport Med..

[2] Fawcett W.J., Haxby E.J., Male D.A. (1999). Magnesium: Physiology and pharmacology. Br. J. Anaesth..

[3] Ahmed F., Mohammed A. (2019). Magnesium: The forgotten electrolyte—A review on hypomagnesemia. Med. Sci..

[4] De Baaij J.H., Hoenderop J.G., Bindels R.J. (2015). Magnesium in man: Implications for health and disease. Physiol. Rev..

[5] Bairoch A. (2000). The ENZYME database in 2000. Nucleic Acids Res..

[6] Caspi R., Altman T., Dreher K., Fulcher C.A., Subhraveti P., Keseler I.M., Kothari A., Krummenacker M., Latendresse M., Mueller L.A. (2012). The MetaCyc database of metabolic pathways and enzymes and the BioCyc collection of pathway/genome databases. Nucleic Acids Res..

[7] Bara M., Guiet-Bara A., Durlach J. (1993). Regulation of sodium and potassium pathways by magnesium in cell membranes. Magnes. Res..

[8] Laban E., Charbon G.A. (1986). Magnesium and cardiac arrhythmias: Nutrient or drug?. J. Am. Coll. Nutr..

[9] Zwillinger L. (1935). Uber die magnesiumwirking auf das. Herz Klin Wochenschr..

[10] Ford E.S., Mokdad A.H. (2003). Dietary magnesium intake in a national sample of US adults. J. Nutr..

[11] Fulgoni V.L., Keast D.R., Bailey R.L., Dwyer J. (2011). Foods, fortificants, and supplements: Where do Americans get their nutrients?. J. Nutr..

[12] Moshfegh A.J., Rhodes D.G., Baer D.J., Murayi T., Clemens J.C., Rumpler W.V., Paul D.R., Sebastian R.S., Kuczynski K.J., Ingwersen L.A. (2008). The US Department of Agriculture Automated Multiple-Pass Method reduces bias in the collection of energy intakes. Am. J. Clin. Nutr..

[13] Papanikolaou Y., Fulgoni V.L. (2018). Grains contribute shortfall nutrients and nutrient density to older US adults: Data from the National Health and Nutrition Examination Survey, 2011–2014. Nutrients.

[14] McNair P., Christensen M.S., Christiansen C., Madsbad S., Transbol I. (1982). Renal hypomagnesaemia in human diabetes mellitus: Its relation to glucose homeostasis. Eur. J. Clin. Invest..

[15] Pham P.C., Pham P.M., Pham P.A., Pham S.V., Pham H.V., Miller J.M., Yanagawa N., Pham P.T. (2005). Lower serum magnesium levels are associated with more rapid decline of renal function in patients with diabetes mellitus type 2. Clin. Nephrol..

[16] Gommers L.M., Hoenderop J.G., Bindels R.J., de Baaij J.H. (2016). Hypomagnesemia in type 2 diabetes: A vicious circle?. Diabetes.

[17] Goldman L., Schafer A.I. (2015). Goldman-Cecil Medicine.

[18] Dasgupta A., Sarma D., Saikia U.K. (2012). Hypomagnesemia in type 2 diabetes mellitus. Indian J. Endocrinol. Metab..

[19] Cheungpasitporn W., Thongprayoon C., Qian Q. (2015). Dysmagnesemia in hospitalized patients: Prevalence and prognostic importance. Mayo Clin. Proc..

[20] Hayes J.P., Ryan M.F., Brazil N., Riordan T.O., Walsh J.B., Coakley D. (1989). Serum hypomagnesaemia in an elderly day-hospital population. Ir. Med. J..

[21] Wong E.T., Rude R.K., Singer F.R., Shaw S.T. (1983). A high prevalence of hypomagnesemia and hypermagnesemia in hospitalized patients. Am. J. Clin. Pathol..

[22] Whang R., Oei T.O., Aikawa J.K., Watanabe A., Vannatta J., Fryer A., Markanich M. (1984). Predictors of clinical hypomagnesemia. Hypokalemia, hypophosphatemia, hyponatremia, and hypocalcemia. Arch. Intern. Med..

[23] Reinhart R.A., Desbiens N.A. (1985). Hypomagnesemia in patients entering the ICU. Crit. Care Med..

[24] Ryzen E., Wagers P.W., Singer F.R., Rude R.K. (1985). Magnesium deficiency in a medical ICU population. Crit. Care Med..

[25] Altura B.M., Altura B.T. (1996). Role of magnesium in patho-physiological processes and the clinical utility of magnesium ion selective electrodes. Scand. J. Clin. Lab. Invest..

[26] Thode J., Juul-Jorgensen B., Seibaek M., Elming H., Borresen E., Jordal R. (1998). Evaluation of an ionized magnesium-pH analyzer—NOVA 8. Scand. J. Clin. Lab. Invest..

[27] Yeh D.D., Chokengarmwong N., Chang Y., Yu L., Arsenault C., Rudolf J., Lee-Lewandrowski E., Lewandrowski K. (2017). Total and ionized magnesium testing in the surgical intensive care unit-Opportunities for improved laboratory and pharmacy utilization. J. Crit. Care.

[28] Altura B.T., Wilimzig C., Trnovec T., Nyulassy S., Altura B.M. (1994). Comparative effects of a Mg-enriched diet and different orally administered magnesium oxide preparations on ionized Mg, Mg metabolism and electrolytes in serum of human volunteers. J. Am. Coll. Nutr..

[29] Rooney M.R., Rudser K.D., Alonso A., Harnack L., Saenger A.K., Lutsey P.L. (2020). Circulating ionized magnesium: Comparisons with circulating total magnesium and the response to magnesium supplementation in a randomized controlled trial. Nutrients.

[30] Blancquaert L., Vervaet C., Derave W. (2019). Predicting and Testing Bioavailability of Magnesium Supplements. Nutrients.

[31] Wilimzig C., Latz R., Vierling W., Mutschler E., Trnovec T., Nyulassy S. (1996). Increase in magnesium plasma level after orally administered trimagnesium dicitrate. European J. Clin. Pharmacol..

[32] Malon A., Brockmann C., Fijalkowska-Morawska J., Rob P., Maj-Zurawska M. (2004). Ionized magnesium in erythrocytes--the best magnesium parameter to observe hypo- or hypermagnesemia. Clin. Chim. Acta.

[33] Greenway D.C., Hindmarsh J.T., Wang J., Khodadeen J.A., Hebert P.C. (1996). Reference interval for whole blood ionized magnesium in a healthy population and the stability of ionized magnesium under varied laboratory conditions. Clin. Biochem..

[34] Palacios C., Wigertz K., Braun M., Martin B.R., McCabe G.P., McCabe L., Pratt J.H., Peacock M., Weaver C.M. (2013). Magnesium retention from metabolic-balance studies in female adolescents: Impact of race, dietary salt, and calcium. Am. J. Clin. Nutr..

[35] Lowenstein F.W., Stanton M.F. (1986). Serum magnesium levels in the United States, 1971–1974. J. Am. Coll. Nutr..

[36] Norbert Wolfgang Tietz (1990). Clinical Guide to Laboratory Tests.

[37] Firoz M., Graber M. (2001). Bioavailability of US commercial magnesium preparations. Magnes. Res..

[38] Von Ehrlich B., Barbagallo M., Classen H.G., Guerrero-Romero F., Mooren F.C., Rodriguez-Moran M., Vierling W., Vormann J., Kisters K. (2017). Significance of magnesium in insulin resistance, metabolic syndrome, and diabetes–recommendations of the Association of Magnesium Research e.V. Trace Elem. Electrolytes.

[39] Costello R.B., Elin R.J., Rosanoff A., Wallace T.C., Guerrero-Romero F., Hruby A., Lutsey P.L., Nielsen F.H., Rodriguez-Moran M., Song Y. (2016). Perspective: The case for an evidence-based reference interval for serum magnesium: The time has come. Adv. Nutr..

[40] Chan K.H., Chacko S.A., Song Y., Cho M., Eaton C.B., Wu W.C., Liu S. (2015). Genetic variations in magnesium-related ion channels may affect diabetes risk among African American and Hispanic American women. J. Nutr..

[41] McHale J. (1997). Thiocyanate interference with Nova’s ionized magnesium electrode. Clin. Chem..

[42] Wong S.L., Epperson A.E., Rogers J., Castro R.J., Jackler R.K., Prochaska J.J. (2020). A multimodal assessment of tobacco use on a university campus and support for adopting a comprehensive tobacco-free policy. Prev. Med..

[43] Rowe S., Alexander N., Clydesdale F.M., Applebaum R.S., Atkinson S., Black R.M., Dwyer J.T., Hentges E., Higley N.A., Lefevre M. (2009). Funding food science and nutrition research: Financial conflicts and scientific integrity. J. Nutr..

